# Association of *APOL1* renal disease risk alleles with *Trypanosoma brucei rhodesiense* infection outcomes in the northern part of Malawi

**DOI:** 10.1371/journal.pntd.0007603

**Published:** 2019-08-14

**Authors:** Kelita Kamoto, Harry Noyes, Peter Nambala, Edward Senga, Janelisa Musaya, Benjamin Kumwenda, Bruno Bucheton, Annette Macleod, Anneli Cooper, Caroline Clucas, Christiane Herz-Fowler, Enock Matove, Arthur M. Chiwaya, John E. Chisi, for the TrypanoGEN Research Group as members of The H3Africa Consortium

**Affiliations:** 1 University of Malawi, College of Medicine, Department of Basic Medical Sciences, Blantyre, Malawi; 2 Centre for Genomic Research, University of Liverpool, United Kingdom; 3 Institut de Recherche pour le Développement (IRD), IRD-CIRAD 177, Montpellier, France; 4 Programme National de Lutte contre la Trypanosomose Humaine Africaine, Conakry, Guinea; 5 Wellcome Trust Centre for Molecular Parasitology, University Place, Glasgow, United Kingdom; 6 Makerere University, Kampala, Uganda; 7 Malawi-Liverpool-Wellcome Trust, Blantyre, Malawi; Insitut Pasteur de Tunis, TUNISIA

## Abstract

*Trypanosoma brucei (T*.*b*.*) rhodesiense* is the cause of the acute form of human African trypanosomiasis (HAT) in eastern and southern African countries. There is some evidence that there is diversity in the disease progression of *T*.*b*. *rhodesiense* in different countries. HAT in Malawi is associated with a chronic haemo-lymphatic stage infection compared to other countries, such as Uganda, where the disease is acute with more marked neurological impairment. This has raised the question of the role of host genetic factors in infection outcomes. A candidate gene association study was conducted in the northern region of Malawi. This was a case-control study involving 202 subjects, 70 cases and 132 controls. All individuals were from one area; born in the area and had been exposed to the risk of infection since birth. Ninety-six markers were genotyped from 17 genes: *IL10*, *IL8*, *IL4*, *HLA-G*, *TNFA*, *IL6*, *IFNG*, *MIF*, *APOL*, *HLA-A*, *IL1B*, *IL4R*, *IL12B*, *IL12R*, *HP*, *HPR*, and *CFH*. There was a strong significant association with *APOL1* G2 allele (p = 0.0000105, OR = 0.14, CI_95_ = [0.05–0.41], BONF = 0.00068) indicating that carriers of the G2 allele were protected against *T*.*b*. *rhodesiense* HAT. SNP rs2069845 in *IL6* had raw p *<* 0.05, but did not remain significant after Bonferroni correction. There were no associations found with the other 15 candidate genes. Our finding confirms results from other studies that the G2 variant of *APOL1* is associated with protection against *T*.*b*. *rhodesiense* HAT.

## Author summary

Though some work has been done on the genetics of trypanosome infections in animals, relatively little is known about the genetics of human African trypanosomiasis (HAT) infections. To test whether any variants are associated with reduced or increased risk of trypanosomiasis, 96 variants in 17 genes were genotyped in patients diagnosed with *T*. *b*. *rhodesiense* HAT and individuals without the disease in this study. From the 96 variants, only one variant G2 in the *APOL1* gene was found to be strongly associated with protection from trypanosomiasis. The results reported here will contribute to the knowledge of the role of human genetics in disease progression, which could offer opportunities for development of much needed new diagnostics and intervention strategies.

## Introduction

Human African trypanosomiasis (HAT), also known as sleeping sickness, is one of the major neglected infectious diseases. Sleeping sickness is endemic in 36 African countries and over 60 million people are at risk of being infected [1]. HAT is more prevalent in rural areas where health care is scarce and affects mainly individuals of reproductive age, increasing their poverty [2].

HAT is a vector-borne parasitic disease transmitted by tsetse flies of the genus *Glossin*a. It is caused by two subspecies of the single-celled parasite *Trypanosoma brucei*: *T*.*b*. *rhodesiense* found in eastern and southern Africa, with reservoirs in livestock and wildlife, and *T*.*b*. *gambiense* found in central and western Africa, which causes the majority of human cases with the main reservoir being humans [2,3]. Sleeping sickness has two clinical stages; the haemolymphatic stage followed by the meningoencephalitic stage. The two subspecies have different rates of disease progression; *T*.*b*. *rhodesiense* infection is typically described as an acute disease with rapid progression to late stage and *T*.*b*. *gambiense* progresses more slowly [3].

Untreated HAT infections are believed to be 100% fatal, with death occurring within weeks or months of symptoms first appearing [4,5]. However, there is increasing evidence that infection by *T*.*b*. *rhodesiense* can result in a wide range of clinical outcomes in its human host [6–8]. Furthermore, there is evidence that individuals from non-endemic areas suffer a more severe infection than people from endemic areas [9,10]. Similar variation in disease progression is also observed in infections with *T*.*b*. *gambiense* [11,12]. Some infected people in Guinea and Côte d’Ivoire progressed to self-cure after refusing treatment, and other individuals in endemic foci in West Africa have shown trypanotolerance analogous to that observed in some West African cattle breeds and in mouse models [13–18].

Genetic polymorphisms in *T*. *b*. *gambiense* as well as the human host have been shown to contribute to different responses to infection [19–21]. Genes involved in immune responses and regulating immunity play important roles in infection outcomes. One such gene is *Apo-lipoprotein-L1* (*APOL1*) whose variants G1 and G2 are associated with kidney disease in African Americans and have been predicted to have been selected because they provide protection against HAT [22,23]. *APOL1* lyses trypanosomes by depolarizing the parasite lysosomal membrane, which leads to osmotic swelling and rupture of the lysosome and then lysis of the trypanosome [24–28]. *Trypanosoma brucei rhodesiense* can infect humans because they express the serum-resistance-associated (*SRA*) protein, which binds to the *SRA*-interacting domain of *APOL1* resulting in the loss of *APOL1* lytic function [24–30]. It has been shown that serum containing G1 and G2 alleles of *APOL1* is lytic to *T*.*b*. *rhodesiense in vitro*, whilst the parasites are resistant to serum containing the G0 allele [22], but evidence that these alleles of *APOL1* mediate resistance to parasites *in vivo* is less conclusive. The G2 allele has been associated with protection against *T*.*b*. *rhodesiense* HAT in one study in Uganda but not in another, and no associations have been found between carriage of the G1 allele and reduced risk of developing *T*.*b*. *rhodesiense* HAT [31,32].

Other genes have also been implicated in the response to infection with *T*. *brucei spp*. Two candidate gene association studies in the Democratic Republic of Congo (DRC) have shown association with the disease and alleles of four genes *(IL6*, *HLA-G*, *IL10* and *APOL1)* out of the 10 genes (*IL1A*, *IL4*, *IL6*, *IL8*, *IL10*, *TNFA*, *IFNG*, *HLA-G*, *HPR* and *APOL1)* studied [21,33]. In other studies, cytokine levels have shown significant association with HAT infections, but the genetic factors regulating this response have not been identified [8,33–37]. In the present study, we investigated the role of single nucleotide polymorphisms in 17 genes (*IL10*, *IL8*, *IL4*, *HLA-G*, *TNFA*, *IL6*, *IFNG*, *MIF*, *APOL1 HLA-A*, *IL1B*, *IL4R*, *IL12B*, *IL12R*, *HP*, *HPR* and *CFH*) for association with susceptibility to HAT using a case-control study design on subjects from the northern part of Malawi.

## Materials and methods

### Population and study design

**Fig 1:**
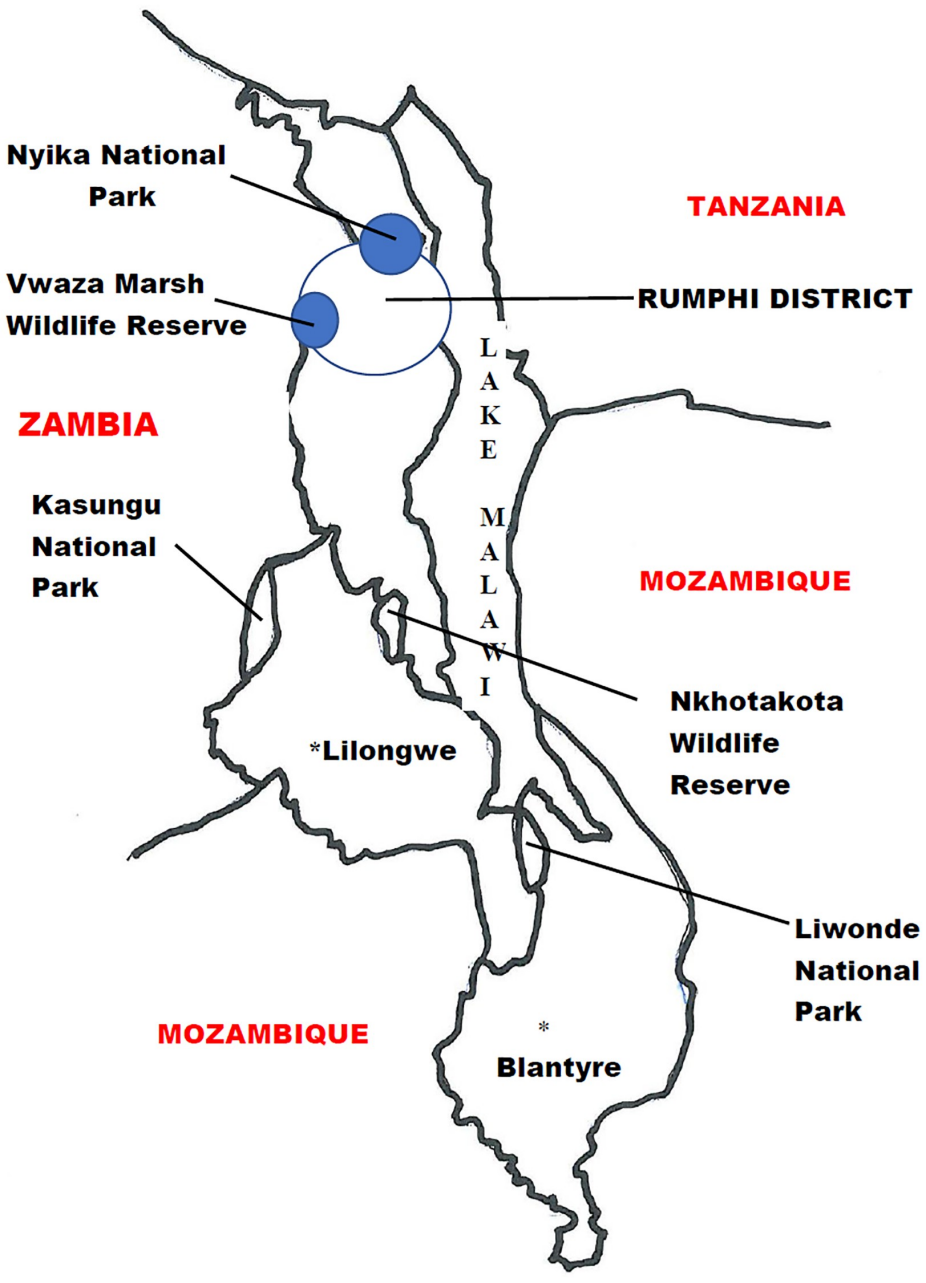
Map of Malawi showing Rumphi District.

The samples came from the Rumphi District in northern Malawi (Fig 1), where the prevalence of HAT cases is highest in Malawi. Between 2000 and 2006, 150 people were confirmed to have died of HAT in Rumphi district alone [38,39].

Cases were identified through active and passive surveys. Hospital case files were checked for previously diagnosed HAT patients and were followed up in their communities. All these cases had been treated with Suramin I.V 20 mg/kg body weight for 23 days and Melarsoprol I.V 3.6 mg/kg body weight for 23 days for the early and late stage of HAT respectively according to Malawi HAT Treatment Guidelines S1 Table. They were then followed up at 3, 6, and 12 months after discharge for review S1 Table. The active screening was conducted during the follow-up of HAT cases. After taking a history and an examination related to HAT infection, venous blood was collected in heparinized tubes and taken to the laboratory at a temperature of 4°C. Eight capillary tubes were prepared from each sample and the buffy coat was examined by microscopy. Out of 350 people screened, 243 individuals entered the study [40]. Cases were defined as individuals in whom trypanosomes were detected by microscopy in blood, lymphatic fluid or cerebral spinal fluids (CSF). Controls were defined as individuals with no signs and symptoms suggestive of HAT and no trypanosomes detected from the blood.

A total of 202 samples, 70 cases and 132 controls were genotyped. Cases and controls came from the same area, were born in the area and had been exposed to infection since birth.

### Ethics statement

The protocol was approved by the Malawi National Health Sciences Research Committee, protocol numbers **NHSRC 15/4/1399** and **Malawi 1213**. There was also local involvement of all stakeholders, and local leaders gave approval for the study to be carried out in the area. All individuals enrolled in the study were 18 years of age or older. All individuals signed informed consent forms in their native language.

### Power analysis

This study was one of six studies of populations of HAT endemic areas in Cameroon, Cote d’Ivoire, Guinea, Malawi, Democratic Republic of Congo (DRC), and Uganda. The studies were designed to have: 80% power to detect odds ratios (OR) >2 for loci with disease allele frequencies of 0.15–0.65; and 100 cases; 100 controls with the 96 SNPs genotyped.

This study had 132 controls, 70 cases from our study area, and had 80% power to detect an OR >2 with disease allele frequencies of 0.1–0.75 with the 96 SNPs genotyped. Power calculations were undertaken using the genetics analysis package gap in R [41–43].

### DNA extraction

DNA was extracted from whole blood (collected in heparin vacutainer tubes (BD) during survey), using Qiagen DNeasy Blood & Tissue Kit (Crawley, UK) as per the manufacturer’s instructions. Extracted DNA samples were temporarily stored at -20°C.

### Genes selection

Genes were selected based on prior knowledge of their role in the development of HAT. The following genes were selected: *IL10*, *IL8*, *IL4*, *HLAG*, *TNFA*, *IL6*, *IFNG*, *MIF*, *APOL*, *HLAA*, *IL1B*, *IL4R*, *IL12B*, *IL12R*, *HP*, *HPR*, and *CFH* [20–22,31,33,36,44–53].

### SNP selection

Ninety-six SNPs were selected for genotyping using two strategies: 1) SNPs that had been previously reported to be associated with HAT or 2) by scanning for sets of linked marker SNP (r^2^ <0.5) across each of *IL4*, *IL8*, *IL6*, *HLAG*, *MIF* and *IFNG*. The SNPs in this second group of genes were selected using a merged SNP dataset obtained from low fold coverage (8-10x) whole genome shotgun data generated from 230 residents living in regions (DRC, Guinea Conakry, Ivory Coast and Uganda) where trypanosomiasis is endemic (TrypanoGEN consortium, sequences at European Nucleotide Archive Study: EGAS00001002602) and 1000 Genomes Project data from African populations [54]. PLINK v1.9 package (https://www.cog-genomics.org/plink/1.9/) [55] was used to estimate linkage disequilibrium (LD) (r^2^) between loci and all sets of SNPs covering the gene were identified. Loci that were excluded during assay development or failed to be genotyped were not replaced and hence not all regions of each gene were linked to marker SNP. S2 Table shows the candidate genes and SNPs selected for this study.

### SNP genotyping

Samples were genotyped by two commercial service providers: INRA- Site de Pierroton, Plateforme Genome Transcriptome de Bordeaux, France and LGC Genomics, Hoddesden, UK. At INRA, two sets of 40 SNP assays were designed using Assay Design Suite v2.0 (Agena Biosciences). SNPs were genotyped with the iPLEX Gold genotyping kit (Agena Biosciences) for the MassArray iPLEX genotyping assay, following the manufacturer’s instructions. Products were detected on a MassArray mass spectrophotometer and data were acquired in real time with MassArray RT software (Agena Biosciences). SNP clustering and validation was carried out with Typer 4.0 software (Agena Biosciences). LGC Genomics genotyped all SNPs that failed genotyping at INRA and some additional SNPs using the PCR based KASP assay [56].

### Data analysis

Plink 1.9 [55] was used for data analysis and R version 3.3.1 (2016-06-21)—"Bug in Your Hair" was used for data visualization (R Foundation for Statistical Computing, Vienna Austria). The data from genotyping were converted to PLINK format and were tested for data completeness, allele frequencies, LD, and Hardy-Weinberg equilibrium (HWE). Individuals and SNP loci with more than 15% and 20% missing data respectively, were removed from the analysis. Fisher’s exact test [57] in PLINK was used for testing the association of SNPs with HAT. One of each pair of SNPs with post genotyping linkage r^2^ > 0.5 were excluded. This increased the power of analysis by reducing the number of tests. Multiple testing was corrected for using a Bonferroni corrected p-value of 0.00077 (0.05/65) [58] and the Benjamini Hochberg false discovery rate (FDR) was used to estimate the probability that the null hypothesis of no association should not be rejected [58,59].

## Results

Two hundred and two samples were sent for genotyping. There were 143 males (70%) and 59 females (30%) with 70 cases (35%) and 132 (65%) controls. The mean ages of the cases and controls were 45 and 41 respectively.

Ninety-six SNPs were genotyped from 17 genes (see **Plink MAP** and **PED files** S1 and S2 Data). After the data was cleaned, 26 individuals with more than 15% missing data were filtered out leaving 176; 59 cases and 117 controls. Nine SNPs with more than 20% missing data were filtered out leaving 87 (see S1 and S2 Figs). Four SNPs, which were not in HWE, were removed. A cut-off of HWE p-value of 1 x 10−8 was used and genotype scatter plots were checked for allele clusters.

To increase the power of analysis, 18 SNPs, which were linked to each other (r^2^ > 0.5), were excluded by pruning (by working across the loci in windows of five SNPs moving one SNP at a time and excluding one of each pair of SNPs with LD greater than r^2^ = 0.5). After quality control and linkage pruning, 65 SNPs were left for association analysis. See S3 Table showing filtered data, and S4 Table showing pruned SNPs.

Results of case-control studies can be confounded by population structure. Most of the cases and controls (95%) were Tumbuka speakers, however there were speakers of five other languages in the cases (3) and controls (8) (Table 1). If the minor language speakers had different allele frequencies from the Tumbuka, this could affect the results.

**Table 1:** Numbers of speakers of each language represented in the sample.

Language	Cases	Controls	Total
Tumbuka	67	124	191
Chewa	1	4	5
Senga	1	0	1
Ngoni	1	2	3
Sena	0	1	1
Lomwe	0	1	1
Total	70	132	202

### Association study

The Fisher’s exact test was used to compare allele frequencies in cases and controls. Allele frequencies differed at two SNPs in two genes (*APOL1* and *IL6*) between the cases and controls. However, only rs71785313 (G2) in *APOL1* (OR 0.14) remained significant after Bonferroni correction (threshold p = 0.00077) and after Benjamini-Hochberg FDR correction, as shown in Table 2. Complete results for all loci are shown in S5 Table. The data was also analysed using logistic regression with gender and age as covariates, neither of these covariates had significant effects (p > 0.05) (see S6 Table).

**Table 2:** Association analysis between HAT cases and controls showing SNPs with lowest p-Values and SNPs in APOL1 gene.

CHR	SNP	GENE	BP	A1	F_A	F_U	A2	P	OR	L95	U95	p(HWE)	BONF	FDR_BH
22	rs71785313	APOL1_G2	36662046	DEL	0.0339	0.1974	INS	1.05E-05**	0.1427	0.05	0.41	1	0.000685	0.000685
7	rs2069845	IL6	22770149	G	0.2458	0.3491	A	0.04512*	0.6074	0.37	1.0	0.8378	1	0.9519
22	rs136177	APOL1	36661842	G	0.06034	0.03947	A	0.3508	1.563	0.57	4.3	1	1	0.9519
22	rs73885319	APOL1_G1	36661906	G	0.1271	0.1207	A	0.7977	1.061	0.54	2.07	1	1	0.9519
22	rs73885316	APOL1	36661674	A	0.01695	0.01293	C	0.8333	1.316	0.22	7.99	1	1	0.9519
22	rs136174	APOL1	36661536	C	0.02586	0.02212	A	0.8607	1.173	0.28	5.00	1	1	0.9519

Analysis of loci within *APOL1* and *IL6* for association with HAT. CHR: Chromosome number, SNP: single nucleotide polymorphism dbSNP id, BP: Physical position (base-pair in GRCh37), A1: Minor allele, A2: Major allele, F_A: Frequency of allele 1 in cases, F_U: Frequency of allele 1 in controls, P: Exact p-value, BONF: Bonferroni corrected p-value, FDR_BH: false discovery rate, OR: Estimated odds ratio (for A1), CI95: 95% confidence interval of odds ratio, HWE: Hardy-Weinberg Equilibrium p-value

* P-value significant

** Bonferroni correction significan**t**

An association was observed at *APOL1*_*G2* rs71785313 (Table 2) with an odds ratio of 0.14 (95% CI: 0.05 to 0.41, p = 0.00001). This indicates a substantially reduced susceptibility to *T*.*b*. *rhodesiense* infection for individuals that possess a G2 variant. No association was found at *APOL1 G1* rs73885319 with *T*.*b*. *rhodesiense* infection (p = 0.80; Table 2). The remaining 15 genes did not show any statistically significant difference in the allele frequencies between cases and controls as shown in S5 Table.

## Discussion

The study looked at 96 SNPs in seventeen genes to test genetic association with HAT in the northern part of Malawi. The main finding of this study is that the *APOL1* G2 variant was strongly associated with protection against *T*.*b*. *rhodesiense* infection in northern Malawi. This is the first study to show such an association in Malawi. Our study showed a seven-fold reduced susceptibility for individuals possessing the *APOL1* G2 variant. This is consistent with a two-centre study in Uganda and Guinea [31] that found a five-fold reduced susceptibility to *T*.*b*. *rhodesiense* for individuals that possess a single copy of G2 variant but no association with the G1 haplotype and *T*.*b*. *rhodesiense*. However, another study in Uganda found no association between the G2 allele and *T*.*b*. *rhodesiense* HAT [32]. The two studies in Uganda were conducted in two very different populations. Cooper et al. [31] found an association in a population from Kabermaido District of mixed Nilotic and Bantu descent with a *G2* allele frequency in controls of 14.4%, whereas Kimuda and colleagues [32] found no association in a population of Bantu descent in Busoga district with a G2 frequency of 8.6%. The *G2* frequency in this study was 19.7% (Table 2), which is comparable to that in the Kabermaido population in Uganda. However, the Malawi population is also of Bantu descent and is linguistically and possibly genetically closer to the Busoga population with low G2 frequency and no association with HAT. Thus, G2 frequencies and association between G2 and HAT do not correlate with the major ethno-linguistic groups. This discrepancy may be due to random genetic drift or specific selection by HAT and/or other diseases at this locus or to variation in the *SRA* gene in the different foci.

There was no association between the G1 allele and HAT in Malawi, which is consistent with both previous studies on *T*.*b*. *rhodesiense* HAT in Uganda [31,32], but this is in contrast to studies of *T*.*b*. *gambiense* HAT population in Guinea where the G1 allele was protective [31,60]. An *in vitro* study also showed that G1 alleles are associated with less lytic potential than G2 alleles [22].

The seven-fold reduced susceptibility for individuals with *APOL1* G2 variant is consistent with the *in vitro* evidence of lysis of *T*.*b*. *rhodesiense* by plasma containing the *APOL1* G2 allele and a study that showed that mice with *APOL1* G2 survived longer after infection with *T*.*b*. *rhodesiense* [22,61]. Both the G1 and G2 renal risk variants are in the *SRA*-interacting domain of *APOL1*. The two-amino acid deletion in G2 rs71785313 prevents the binding of *SRA* to *APOL1* [22,61,62], enabling carriers of the G2 variant to lyse the parasites.

The G1 haplotype consists of two missense mutations in almost perfect linkage disequilibrium (rs73885319 and rs60910145). In this study, only rs73885319 was genotyped (Table 2 and S2 Table), but no association was found with HAT in Malawi.

In conclusion, this study has shown that host genetic polymorphisms play a role in the control of infections and morbidity in HAT. Of the 17 genes studied, only the *APOL1* G2 variant showed a statistically significant association with *T*. *b*. *rhodesiense* infections after Bonferroni correction for multiple testing. This is the first study in Malawi to show this association and increases support for a role for this allele in disease resistance which has previously been found associated in one study but not associated in another study. Further studies will be required to determine the effect of the G1 variant on the severity of *T*. *b*. *rhodesiense* infections and gene expression between the cases and the controls.

## Supporting information

S1 Data The TrypanoGEN Malawi samples MAP file. (TXT)

S2 Data The TrypanoGEN Malawi samples PED file. (TXT)

S1 Fig Individuals with missing data. (TIF)

S2 Fig SNPs with missing data. (TIF)

S1 Table Malawi HAT Treatment Guidelines. (DOCX)

S2 Table Candidate genes and SNPs and amino acid change. (DOCX)

S3 Table Filtered data. (DOCX)

S4 Table Showing 18 pruned SNPs in bold. (DOCX)

S5 Table Association analysis between HAT cases and controls of 65 SNPs. (DOCX)

S6 Table Malawi association tests using logistic regression looking at effect of age, sex, and district-on HAT. (DOCX)
