# Supplementary figures and images for: Association of APOL1 renal disease risk alleles with Trypanosoma brucei rhodesiense infection outcomes in the northern part of Malawi
Source: PLoS Negl Trop Dis. 2019 Aug 14;13(8):e0007603. doi: 10.1371/journal.pntd.0007603 (PMC6750591; doi:10.1371/journal.pntd.0007603)

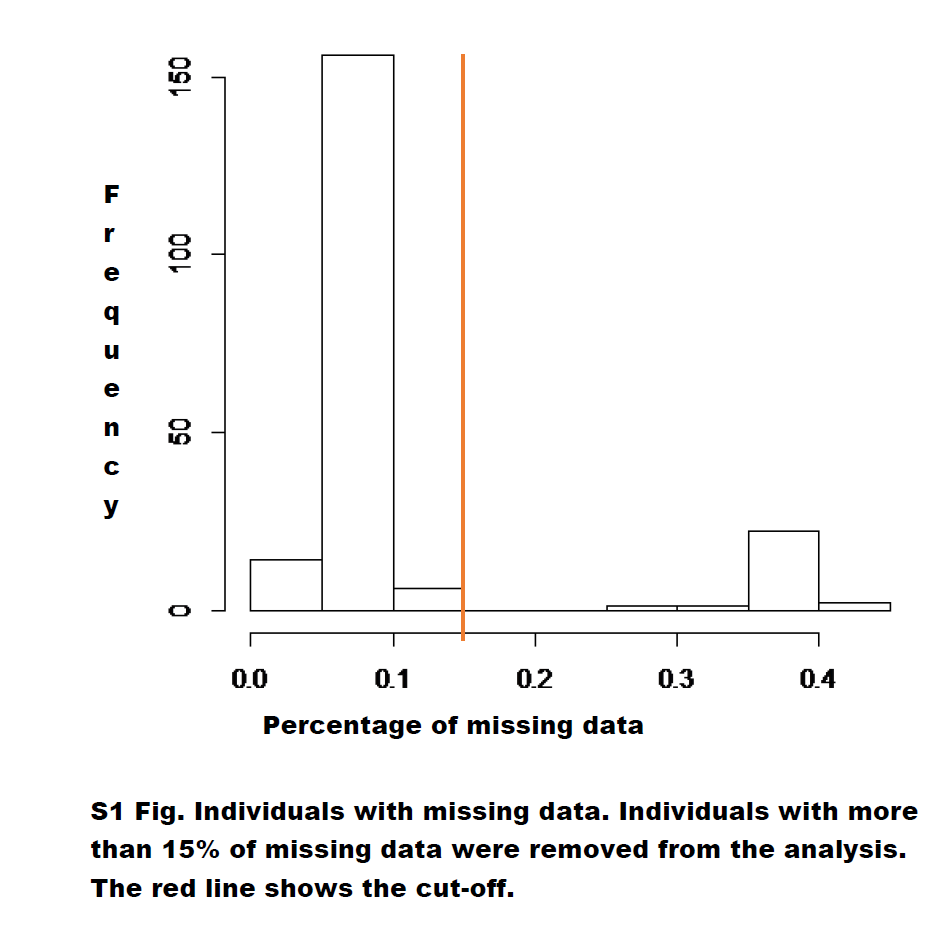

Supplement: S1 Fig — (TIF) [file pntd.0007603.s003.tif]

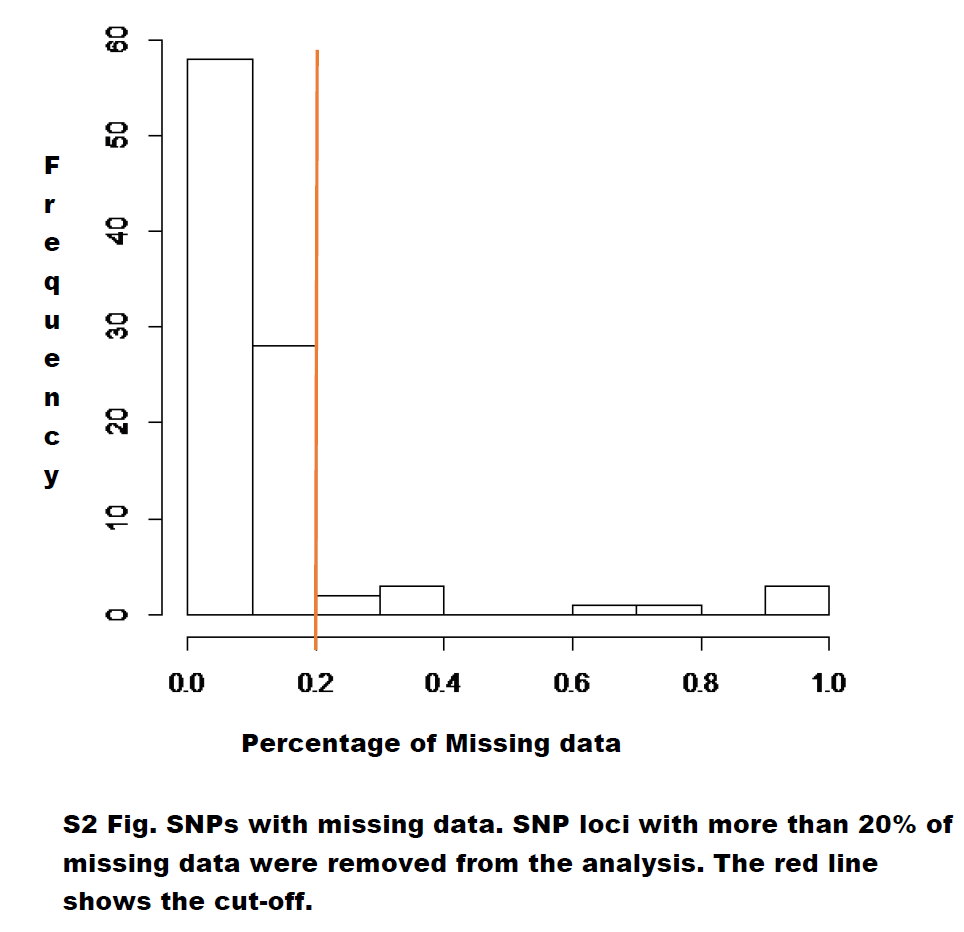

Supplement: S2 Fig — (TIF) [file pntd.0007603.s004.tif]
