# Supplementary material for: Association of APOL1 renal disease risk alleles with Trypanosoma brucei rhodesiense infection outcomes in the northern part of Malawi
Source: PLoS Negl Trop Dis. 2019 Aug 14;13(8):e0007603. doi: 10.1371/journal.pntd.0007603 (PMC6750591; doi:10.1371/journal.pntd.0007603)
Supplement: S1 Table — (DOCX) [file pntd.0007603.s005.docx]

**S1 Table. Malawi Hat treatment guidelines**


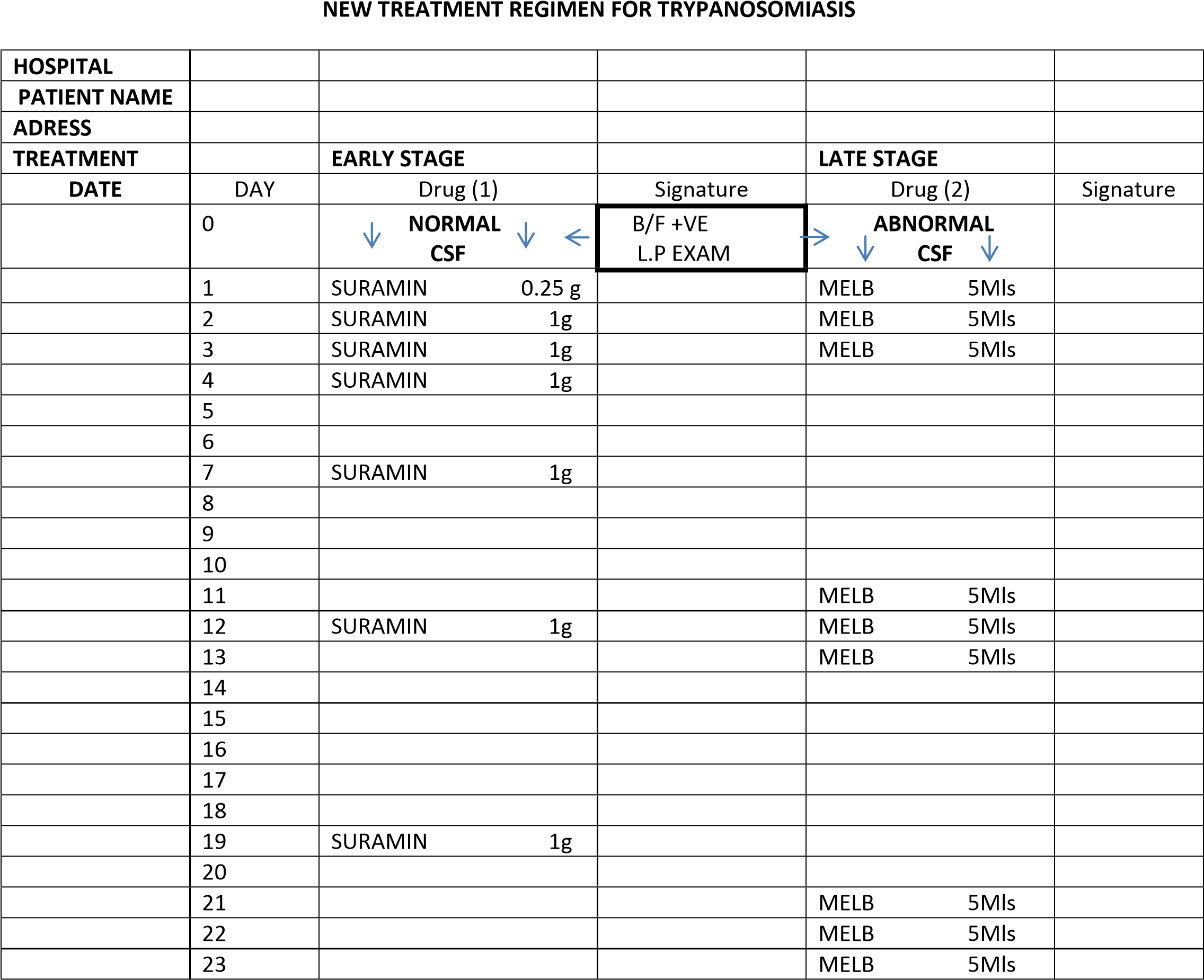


**STAGE DETERMINATION:**

Lumbar puncture should be done on day “O” in order to stage the disease before treatment

If any reaction to the drug is observed (skin exfoliative dermatitis, rash or reactive encephalitis), stop treatment immediately and inform the Clinical Officer/Medical Doctor on duty

SURAMIN: DOSE: 20 mg/kg body weight

Suramin must always be freshly prepared with sterile water for injection to make a 10% solution

MELARSOPROL: DOSE: 3.6 mg/kg body weight

STRICTLY (adult maximum dose: 180 mg + 1 ampoule=5mls). Melarsoprol can be given IV. push but avoid tissueing because the drug is highly irritant.

FOLLOW UP: Review the patient at 3, 6, and 12 months after discharge

STEROIDS: Corticosteroids should not be administered as a routine therapy
