# Supplementary material for: Association of APOL1 renal disease risk alleles with Trypanosoma brucei rhodesiense infection outcomes in the northern part of Malawi
Source: PLoS Negl Trop Dis. 2019 Aug 14;13(8):e0007603. doi: 10.1371/journal.pntd.0007603 (PMC6750591; doi:10.1371/journal.pntd.0007603)
Supplement: S3 Table — (DOCX) [file pntd.0007603.s007.docx]

**S3 Table. Filtered data**

| FILTER | FILTER PARAMETER | NUMBER OF SAMPLES (People) | NUMBER OF SNP (Variants) |
| --- | --- | --- | --- |
| None | N/A | 202 | 96 |
| Missing data for each individual | 0.15 | 176  (26 individual removed due missing data) | 96 |
| Missing data for each locus | 0.20 | 176 | 87  ( 9 variants removed due to missing data) |
| Hardy Weinberg (hwe) |  | 176 | 83  (4 variants removed) |
| Remove one pair of loci highly linked to each other linkage greater than r^2^ =0.5 order to increase power. |  | 176 | 65  (18 removed) |

**Variants and Number of Samples have been filtered in order to clean the Data**
