# Supplementary material for: Association of APOL1 renal disease risk alleles with Trypanosoma brucei rhodesiense infection outcomes in the northern part of Malawi
Source: PLoS Negl Trop Dis. 2019 Aug 14;13(8):e0007603. doi: 10.1371/journal.pntd.0007603 (PMC6750591; doi:10.1371/journal.pntd.0007603)
Supplement: S4 Table — (DOCX) [file pntd.0007603.s008.docx]

**S4 Table. Showing 18 Pruned SNPs in bold**

| CHR | SNP | GENE |
| --- | --- | --- |
| 1 | rs1061170 | CFH |
| 1 | rs1800872 | IL10 |
| 2 | rs1143629 | IL1B |
| 4 | rs114259658 | IL8 |
| 4 | rs2227307 | IL8 |
| 4 | rs2227545 | IL8 |
| 4 | rs13112910 | IL8 |
| 4 | rs58478511 | IL8 |
| **4** | **rs62312369** | **IL8** |
| 5 | rs2243250 | IL4 |
| **5** | **rs2070874** | **IL4** |
| 5 | rs734244 | IL4 |
| 5 | rs2243255 | IL4 |
| 5 | rs2243256 | IL4 |
| 5 | rs2243258 | IL4 |
| 5 | rs2243261 | IL4 |
| **5** | **rs71889624** | **IL4** |
| 5 | rs2243268 | IL4 |
| 5 | rs9282745 | IL4 |
| 5 | rs2243270 | IL4 |
| 5 | rs2243279 | IL4 |
| **5** | **rs2243282** | **IL4** |
| 5 | rs73269366 | IL4 |
| 5 | rs3212227 | IL12B |
| 5 | rs2546890 | IL12B |
| 6 | rs1736936 | HLAG |
| **6** | **rs17875389** | **HLAG** |
| 6 | rs1130363 | HLAG |
| 6 | rs371194629 | HLAG |
| **6** | **rs17179108** | **HLAG** |
| **6** | **rs9380142** | **HLAG** |
| 6 | rs1610696 | HLAG |
| 6 | rs1233330 | HLAG |
| 6 | rs1611139 | HLAG |
| 6 | rs2517898 | HLAG |
| 6 | rs141206123 | HLAG |
| **6** | **rs2517897** | **HLAG** |
| **6** | **rs12662618** | **HLAG** |
| **6** | **rs1136754** | **HLAA** |
| 6 | rs1059563 | HLAA |
| 6 | rs1059564 | HLAA |
| 6 | rs1800630 | TNFA |
| 6 | rs1800629 | TNFA |
| 7 | rs62449495 | IL6 |
| 7 | rs2069830 | IL6 |
| 7 | rs2069834 | IL6 |
| **7** | **rs2069837** | **IL6** |
| 7 | rs1474347 | IL6 |
| 7 | rs2066992 | IL6 |
| **7** | **rs2069842** | **IL6** |
| 7 | rs1548216 | IL6 |
| 7 | rs2069843 | IL6 |
| 7 | rs2069845 | IL6 |
| 7 | rs2069855 | IL6 |
| 7 | rs1818879 | IL6 |
| 12 | rs2069728 | IFNG |
| 12 | rs2069723 | IFNG |
| **12** | **rs2069720** | **IFNG** |
| 12 | rs2069718 | IFNG |
| 12 | rs1861493 | IFNG |
| 12 | rs2069713 | IFNG |
| 12 | rs2430561 | IFNG |
| 12 | rs78554979 | IFNG |
| 12 | rs2069705 | IFNG |
| 16 | rs1801275 | IL4R |
| 16 | rs1424241 | HPR |
| 16 | rs8062041 | HP |
| **16** | **rs7185840** | **HPR** |
| 16 | rs2021171 | HPR |
| **16** | **rs152828** | **HPR** |
| 19 | rs375947 | IL12RB1 |
| 19 | rs11575934 | IL12RB1 |
| 22 | rs12483859 | MIF |
| **22** | **rs36086171** | **MIF** |
| 22 | rs9282783 | MIF |
| **22** | **rs11548056.** | **MIF** |
| 22 | rs35235644 | MIF |
| 22 | rs2000466 | MIF |
| 22 | rs34383331 | MIF |
| 22 | rs136174 | APOL1 |
| 22 | rs73885316 | APOL1 |
| **22** | **rs136177** | **APOL1** |
| 22 | rs73885319 | APOL1 |
| **22** | **rs143830837** | **APOL1** |
| 22 | rs71785313 | APOL1G2 |

**Prunned SNPs from pairs which had linkage greater than r^2^ =0.5**
