# Supplementary material for: Association of APOL1 renal disease risk alleles with Trypanosoma brucei rhodesiense infection outcomes in the northern part of Malawi
Source: PLoS Negl Trop Dis. 2019 Aug 14;13(8):e0007603. doi: 10.1371/journal.pntd.0007603 (PMC6750591; doi:10.1371/journal.pntd.0007603)
Supplement: S5 Table — (DOCX) [file pntd.0007603.s009.docx]

**S5 Table. Association results of the 65 SNPs with HAT**

| CHR | SNP | GENE | BP | A1 | F_A | F_U | A2 | P | OR | L95 | U95 | P (HWE) | BONF | FDR_BH |
| --- | --- | --- | --- | --- | --- | --- | --- | --- | --- | --- | --- | --- | --- | --- |
| 22 | rs71785313 | APOL1_G2 | 36662046 | DEL | 0.034 | 0.197 | INSERT | 1.05E-05^**^ | 0.1427 | 0.05 | 0.41 | 1 | 0.0006854 | 0.000685 |
| 7 | rs2069845 | IL6 | 22770149 | G | 0.246 | 0.349 | A | 0.04512^*^ | 0.6074 | 0.37 | 1.00 | 0.8378 | 1 | 0.9519 |
| 12 | rs2069728 | IFNG | 68547784 | T | 0.254 | 0.353 | C | 0.06092 | 0.6236 | 0.38 | 1.02 | 1 | 1 | 0.9519 |
| 22 | rs34383331 | MIF | 24238079 | A | 0.339 | 0.246 | T | 0.06696 | 1.575 | 0.97 | 2.56 | 0.207 | 1 | 0.9519 |
| 22 | rs35235644 | MIF | 24237822 | C | 0.136 | 0.082 | G | 0.1107 | 1.759 | 0.87 | 3.56 | 0.5515 | 1 | 0.9519 |
| 22 | rs12483859 | MIF | 24234807 | T | 0.509 | 0.422 | C | 0.1261 | 1.414 | 0.91 | 2.21 | 0.1874 | 1 | 0.9519 |
| 5 | rs734244 | IL4 | 132010726 | T | 0.424 | 0.496 | C | 0.1931 | 0.7483 | 0.48 | 1.17 | 0.000746 | 1 | 0.9519 |
| 5 | rs2243270 | IL4 | 132014109 | A | 0.195 | 0.254 | G | 0.2069 | 0.7099 | 0.41 | 1.22 | 0.6248 | 1 | 0.9519 |
| 4 | rs114259658 | IL8 | 74605639 | A | 0.008 | 0.034 | T | 0.2267 | 0.2393 | 0.03 | 1.94 | 1 | 1 | 0.9519 |
| 6 | rs1800629 | TNFA | 31543031 | A | 0.186 | 0.142 | G | 0.2476 | 1.382 | 0.76 | 2.50 | 1 | 1 | 0.9519 |
| 6 | rs2517898 | HLAG | 29799746 | G | 0.364 | 0.306 | C | 0.2537 | 1.3 | 0.81 | 2.08 | 0.126 | 1 | 0.9519 |
| 12 | rs78554979 | IFNG | 68554636 | C | 0.093 | 0.134 | T | 0.2621 | 0.6666 | 0.32 | 1.38 | 1 | 1 | 0.9519 |
| 12 | rs2430561 | IFNG | 68552522 | A | 0.223 | 0.171 | T | 0.2716 | 1.393 | 0.79 | 2.45 | 1 | 1 | 0.9519 |
| 5 | rs2243258 | IL4 | 132012110 | T | 0.127 | 0.091 | C | 0.3109 | 1.463 | 0.72 | 2.96 | 0.5954 | 1 | 0.9519 |
| 16 | rs152828 | HPR | 72123886 | T | 0.009 | 0.004 | C | 0.3295 | 2.044 | 0.13 | 32.98 | 1 | 1 | 0.9519 |
| 22 | rs136177 | APOL1 | 36661842 | G | 0.060 | 0.039 | A | 0.3508 | 1.563 | 0.57 | 4.31 | 1 | 1 | 0.9519 |
| 6 | rs1130363 | HLAG | 29797696 | A | 0.343 | 0.395 | G | 0.3644 | 0.7999 | 0.49 | 1.29 | 0.02662 | 1 | 0.9519 |
| 7 | rs2069834 | IL6 | 22767828 | T | 0.068 | 0.095 | C | 0.3702 | 0.6942 | 0.30 | 1.61 | 1 | 1 | 0.9519 |
| 5 | rs2243279 | IL4 | 132016227 | A | 0.110 | 0.082 | G | 0.3818 | 1.388 | 0.66 | 2.92 | 1 | 1 | 0.9519 |
| 7 | rs62449495 | IL6 | 22764338 | A | 0.042 | 0.065 | G | 0.4015 | 0.6401 | 0.23 | 1.81 | 1 | 1 | 0.9519 |
| 16 | rs1424241 | HPR | 72078907 | A | 0.110 | 0.138 | G | 0.4512 | 0.7738 | 0.39 | 1.54 | 0.4489 | 1 | 0.9519 |
| 12 | rs2069723 | IFNG | 68548594 | C | 0.017 | 0.009 | T | 0.4562 | 1.931 | 0.27 | 13.88 | 1 | 1 | 0.9519 |
| 6 | rs1611139 | HLAG | 29799116 | T | 0.236 | 0.275 | G | 0.4667 | 0.8151 | 0.48 | 1.39 | 0.007602 | 1 | 0.9519 |
| 12 | rs1861493 | IFNG | 68551196 | G | 0.051 | 0.034 | A | 0.4819 | 1.5 | 0.51 | 4.43 | 1 | 1 | 0.9519 |
| 5 | rs2243268 | IL4 | 132013963 | C | 0.331 | 0.366 | A | 0.5172 | 0.8538 | 0.54 | 1.36 | 1 | 1 | 0.9519 |
| 7 | rs2069843 | IL6 | 22769994 | A | 0.102 | 0.125 | G | 0.5417 | 0.7925 | 0.39 | 1.62 | 0.6883 | 1 | 0.9519 |
| 7 | rs1818879 | IL6 | 22772727 | A | 0.110 | 0.134 | G | 0.5537 | 0.8028 | 0.40 | 1.60 | 1 | 1 | 0.9519 |
| 7 | rs2069842 | IL6 | 22769310 | A | 0.051 | 0.069 | G | 0.5662 | 0.7232 | 0.28 | 1.90 | 1 | 1 | 0.9519 |
| 5 | rs2546890 | IL12B | 158759900 | A | 0.305 | 0.276 | G | 0.574 | 1.15 | 0.71 | 1.87 | 0.6384 | 1 | 0.9519 |
| 5 | rs2243255 | IL4 | 132011737 | A | 0.129 | 0.155 | G | 0.5746 | 0.8105 | 0.42 | 1.55 | 0.1378 | 1 | 0.9519 |
| 1 | rs1061170 | CFH | 196659237 | C | 0.407 | 0.435 | T | 0.6076 | 0.8914 | 0.57 | 1.40 | 0.1876 | 1 | 0.9519 |
| 6 | rs17875389 | HLAG | 29794484 | G | 0.017 | 0.030 | A | 0.6079 | 0.5542 | 0.11 | 2.71 | 0.001967 | 1 | 0.9519 |
| 16 | rs1801275 | IL4R | 27374400 | A | 0.203 | 0.181 | G | 0.6151 | 1.155 | 0.66 | 2.02 | 0.1164 | 1 | 0.9519 |
| 7 | rs2069837 | IL6 | 22768027 | G | 0.203 | 0.185 | A | 0.6176 | 1.122 | 0.64 | 1.96 | 0.3557 | 1 | 0.9519 |
| 5 | rs2243261 | IL4 | 132012806 | T | 0.093 | 0.082 | G | 0.6185 | 1.152 | 0.53 | 2.51 | 1 | 1 | 0.9519 |
| 5 | rs2243256 | IL4 | 132011753 | DEL | 0.119 | 0.103 | T | 0.6527 | 1.167 | 0.58 | 2.35 | 1 | 1 | 0.9519 |
| 6 | rs1059564 | HLAA | 29911930 | T | 0.000 | 0.004 | C | 0.6657 | 0 | 0.00 | nan | 1 | 1 | 0.9519 |
| 5 | rs3212227 | IL12B | 158742950 | G | 0.348 | 0.371 | T | 0.6818 | 0.904 | 0.57 | 1.44 | 1 | 1 | 0.9519 |
| 16 | rs7185840 | HPR | 72102112 | A | 0.144 | 0.164 | G | 0.7004 | 0.8593 | 0.46 | 1.60 | 0.07922 | 1 | 0.9519 |
| 7 | rs2066992 | IL6 | 22768249 | T | 0.061 | 0.055 | G | 0.7132 | 1.134 | 0.43 | 2.96 | 0.2715 | 1 | 0.9519 |
| 12 | rs2069720 | IFNG | 68549710 | T | 0.059 | 0.069 | C | 0.7358 | 0.8514 | 0.34 | 2.13 | 0.08466 | 1 | 0.9519 |
| 6 | rs1233330 | HLAG | 29799103 | A | 0.068 | 0.082 | G | 0.7553 | 0.8153 | 0.35 | 1.92 | 0.5515 | 1 | 0.9519 |
| 19 | rs11575934 | IL12RB1 | 18186618 | C | 0.093 | 0.086 | T | 0.7677 | 1.09 | 0.50 | 2.36 | 0.1915 | 1 | 0.9519 |
| 12 | rs2069705 | IFNG | 68555011 | G | 0.407 | 0.392 | A | 0.7735 | 1.062 | 0.68 | 1.67 | 0.8464 | 1 | 0.9519 |
| 4 | rs2227307 | IL8 | 74606669 | G | 0.483 | 0.500 | T | 0.7757 | 0.9333 | 0.60 | 1.46 | 0.5705 | 1 | 0.9519 |
| 2 | rs1143629 | IL1B | 113593518 | G | 0.449 | 0.435 | A | 0.7763 | 1.06 | 0.68 | 1.66 | 0.05692 | 1 | 0.9519 |
| 16 | rs8062041 | HPR | 72088964 | C | 0.458 | 0.439 | T | 0.7767 | 1.08 | 0.69 | 1.69 | 1 | 1 | 0.9519 |
| 22 | rs73885319 | APOL1_G1 | 36661906 | G | 0.127 | 0.121 | A | 0.7977 | 1.061 | 0.54 | 2.07 | 1 | 1 | 0.9519 |
| 5 | rs2243283 | IL4 | 132016593 | G | 0.214 | 0.203 | C | 0.8179 | 1.069 | 0.59 | 1.95 | 0.1883 | 1 | 0.9519 |
| 22 | rs73885316 | APOL1 | 36661674 | A | 0.017 | 0.013 | C | 0.8333 | 1.316 | 0.22 | 7.99 | 1 | 1 | 0.9519 |
| 7 | rs2069855 | IL6 | 22772624 | C | 0.017 | 0.017 | T | 0.834 | 0.9828 | 0.18 | 5.45 | 1 | 1 | 0.9519 |
| 5 | rs2243250 | IL4 | 132009154 | C | 0.212 | 0.203 | T | 0.8348 | 1.058 | 0.61 | 1.83 | 0.07988 | 1 | 0.9519 |
| 22 | rs136174 | APOL1 | 36661536 | C | 0.026 | 0.022 | A | 0.8607 | 1.173 | 0.28 | 5.00 | 1 | 1 | 0.9519 |
| 4 | rs13112910 | IL8 | 74609755 | G | 0.381 | 0.370 | A | 0.8615 | 1.052 | 0.67 | 1.66 | 0.6885 | 1 | 0.9519 |
| 12 | rs2069713 | IFNG | 68552476 | C | 0.034 | 0.030 | T | 0.8782 | 1.128 | 0.32 | 3.93 | 1 | 1 | 0.9519 |
| 5 | rs73269366 | IL4 | 132018749 | T | 0.051 | 0.052 | C | 0.8993 | 0.9821 | 0.36 | 2.69 | 0.2588 | 1 | 0.9519 |
| 22 | rs9282783 | MIF | 24236359 | G | 0.059 | 0.060 | C | 0.9062 | 0.982 | 0.39 | 2.50 | 0.3408 | 1 | 0.9519 |
| 5 | rs9282745 | IL4 | 132014000 | A | 0.059 | 0.065 | T | 0.9088 | 0.9123 | 0.36 | 2.30 | 0.3831 | 1 | 0.9519 |
| 6 | rs141206123 | HLAG | 29799849 | C | 0.068 | 0.069 | DEL | 0.9117 | 0.9818 | 0.41 | 2.37 | 0.4258 | 1 | 0.9519 |
| 7 | rs2069830 | IL6 | 22767137 | T | 0.076 | 0.078 | C | 0.9162 | 0.9817 | 0.43 | 2.26 | 0.5104 | 1 | 0.9519 |
| 4 | rs2227545 | IL8 | 74608727 | C | 0.110 | 0.116 | A | 0.9301 | 0.94 | 0.47 | 1.90 | 0.6483 | 1 | 0.9519 |
| 6 | rs1800630 | TNFA | 31542476 | A | 0.119 | 0.121 | C | 0.931 | 0.9808 | 0.50 | 1.94 | 0.2106 | 1 | 0.9519 |
| 6 | rs12662618 | HLAG | 29800211 | C | 0.153 | 0.157 | T | 0.938 | 0.97 | 0.52 | 1.79 | 0.2997 | 1 | 0.9519 |
| 4 | rs58478511 | IL8 | 74610033 | A | 0.307 | 0.311 | G | 0.9505 | 0.9824 | 0.60 | 1.60 | 1 | 1 | 0.9519 |
| 1 | rs1800872 | IL10 | 206946407 | T | 0.322 | 0.323 | G | 0.9519 | 0.9943 | 0.62 | 1.60 | 0.2869 | 1 | 0.9519 |

CHR: Chromosome number, SNP: single nucleotide polymorphism dbSNP id, BP: Physical position (base-pair in GRCh37), A1: Minor allele, A2: Major allele, F_A: Frequency of allele 1 in cases, F_U: Frequency of allele 1 in controls, P: Exact p-value, BONF: Bonferroni corrected p-value, FDR_BH: false discovery rate, OR: Estimated odds ratio (for A1), CI95: 95% confidence interval of odds ratio, HWE: Hardy-Weinberg Equilibrium p-value

* P-value significant

** Bonferroni correction significan**t**
