# Supplementary material for: Association of APOL1 renal disease risk alleles with Trypanosoma brucei rhodesiense infection outcomes in the northern part of Malawi
Source: PLoS Negl Trop Dis. 2019 Aug 14;13(8):e0007603. doi: 10.1371/journal.pntd.0007603 (PMC6750591; doi:10.1371/journal.pntd.0007603)
Supplement: S6 Table — (DOCX) [file pntd.0007603.s010.docx]

**S6 Table. Malawi association results using logistics regression to compare the cases and controls**

| CHR | SNP | GENE | BP | A1 | TEST | NMISS | OR | SE | L95 | U95 | STAT | P |
| --- | --- | --- | --- | --- | --- | --- | --- | --- | --- | --- | --- | --- |
| 1 | rs1061170 | CFH | 196659237 | C | ADD | 197 | 0.7832 | 0.2384 | 0.4909 | 1.25 | -1.025 | 0.3054 |
| 1 | rs1061170 | CFH | 196659237 | C | sex | 197 | 1.023 | 0.3409 | 0.5243 | 1.995 | 0.0657 | 0.9476 |
| 1 | rs1061170 | CFH | 196659237 | C | AGE | 197 | 1.016 | 0.0114 | 0.9937 | 1.039 | 1.41 | 0.1587 |
| 1 | rs1061170 | CFH | 196659237 | C | DISTRICT | 197 | 1.05 | 0.3092 | 0.573 | 1.925 | 0.1586 | 0.874 |
| 2 | rs1143629 | IL1B | 113593518 | G | ADD | 195 | 0.9506 | 0.2117 | 0.6278 | 1.439 | -0.2394 | 0.8108 |
| 2 | rs1143629 | IL1B | 113593518 | G | sex | 195 | 1.093 | 0.3421 | 0.5589 | 2.136 | 0.2592 | 0.7955 |
| 2 | rs1143629 | IL1B | 113593518 | G | AGE | 195 | 1.015 | 0.01143 | 0.9925 | 1.038 | 1.302 | 0.1928 |
| 2 | rs1143629 | IL1B | 113593518 | G | DISTRICT | 195 | 1.037 | 0.3147 | 0.5596 | 1.922 | 0.1154 | 0.9082 |
| 4 | rs114259658 | IL8 | 74605639 | A | ADD | 198 | 0.2103 | 1.077 | 0.0255 | 1.735 | -1.448 | 0.1476 |
| 4 | rs114259658 | IL8 | 74605639 | A | sex | 198 | 1.134 | 0.3472 | 0.5743 | 2.24 | 0.3627 | 0.7168 |
| 4 | rs114259658 | IL8 | 74605639 | A | AGE | 198 | 1.013 | 0.01141 | 0.9909 | 1.036 | 1.159 | 0.2464 |
| 4 | rs114259658 | IL8 | 74605639 | A | DISTRICT | 198 | 1 | 0.3115 | 0.5431 | 1.841 | 0.00021 | 0.9998 |
| 4 | rs2227307 | IL8 | 74606669 | T | ADD | 184 | 0.9223 | 0.2208 | 0.5983 | 1.422 | -0.3662 | 0.7142 |
| 4 | rs2227307 | IL8 | 74606669 | T | sex | 184 | 0.8175 | 0.3682 | 0.3972 | 1.682 | -0.5473 | 0.5842 |
| 4 | rs2227307 | IL8 | 74606669 | T | AGE | 184 | 1.023 | 0.01201 | 0.9995 | 1.048 | 1.921 | 0.05469 |
| 4 | rs2227307 | IL8 | 74606669 | T | DISTRICT | 184 | 1.156 | 0.3288 | 0.607 | 2.203 | 0.4416 | 0.6587 |
| 4 | rs2227545 | IL8 | 74608727 | C | ADD | 198 | 0.7759 | 0.3428 | 0.3963 | 1.519 | -0.7401 | 0.4593 |
| 4 | rs2227545 | IL8 | 74608727 | C | sex | 198 | 1.044 | 0.3405 | 0.5354 | 2.034 | 0.1252 | 0.9004 |
| 4 | rs2227545 | IL8 | 74608727 | C | AGE | 198 | 1.016 | 0.01128 | 0.9936 | 1.038 | 1.388 | 0.165 |
| 4 | rs2227545 | IL8 | 74608727 | C | DISTRICT | 198 | 1.034 | 0.3079 | 0.5653 | 1.89 | 0.1079 | 0.9141 |
| 4 | rs58478511 | IL8 | 74610033 | A | ADD | 190 | 1.017 | 0.2246 | 0.655 | 1.58 | 0.07618 | 0.9393 |
| 4 | rs58478511 | IL8 | 74610033 | A | sex | 190 | 0.8734 | 0.3501 | 0.4397 | 1.735 | -0.3867 | 0.699 |
| 4 | rs58478511 | IL8 | 74610033 | A | AGE | 190 | 1.016 | 0.01138 | 0.9938 | 1.039 | 1.418 | 0.1562 |
| 4 | rs58478511 | IL8 | 74610033 | A | DISTRICT | 190 | 1.076 | 0.3143 | 0.5813 | 1.993 | 0.2343 | 0.8147 |
| 4 | rs62312369 | IL8 | 74610397 | 0 | ADD | 198 | NA | NA | NA | NA | NA | NA |
| 4 | rs62312369 | IL8 | 74610397 | 0 | sex | 198 | NA | NA | NA | NA | NA | NA |
| 4 | rs62312369 | IL8 | 74610397 | 0 | AGE | 198 | NA | NA | NA | NA | NA | NA |
| 4 | rs62312369 | IL8 | 74610397 | 0 | DISTRICT | 198 | NA | NA | NA | NA | NA | NA |
| 5 | rs734244 | IL4 | 132010726 | T | ADD | 195 | 0.944 | 0.1946 | 0.6446 | 1.382 | -0.2964 | 0.767 |
| 5 | rs734244 | IL4 | 132010726 | T | sex | 195 | 1.043 | 0.3415 | 0.534 | 2.037 | 0.123 | 0.9021 |
| 5 | rs734244 | IL4 | 132010726 | T | AGE | 195 | 1.016 | 0.0114 | 0.9937 | 1.039 | 1.408 | 0.1591 |
| 5 | rs734244 | IL4 | 132010726 | T | DISTRICT | 195 | 1.095 | 0.3132 | 0.5926 | 2.022 | 0.289 | 0.7726 |
| 5 | rs2243255 | IL4 | 132011737 | A | ADD | 194 | 1.024 | 0.2961 | 0.5729 | 1.829 | 0.07855 | 0.9374 |
| 5 | rs2243255 | IL4 | 132011737 | A | sex | 194 | 1.054 | 0.3444 | 0.5365 | 2.069 | 0.1519 | 0.8793 |
| 5 | rs2243255 | IL4 | 132011737 | A | AGE | 194 | 1.016 | 0.0114 | 0.9936 | 1.039 | 1.396 | 0.1627 |
| 5 | rs2243255 | IL4 | 132011737 | A | DISTRICT | 194 | 1.001 | 0.3143 | 0.5406 | 1.853 | 0.00282 | 0.9978 |
| 5 | rs2243258 | IL4 | 132012110 | T | ADD | 198 | 1.294 | 0.3452 | 0.658 | 2.546 | 0.7473 | 0.4549 |
| 5 | rs2243258 | IL4 | 132012110 | T | sex | 198 | 1.011 | 0.3439 | 0.5155 | 1.984 | 0.0329 | 0.9738 |
| 5 | rs2243258 | IL4 | 132012110 | T | AGE | 198 | 1.015 | 0.01129 | 0.9932 | 1.038 | 1.352 | 0.1765 |
| 5 | rs2243258 | IL4 | 132012110 | T | DISTRICT | 198 | 1.085 | 0.3128 | 0.5879 | 2.003 | 0.2615 | 0.7937 |
| 5 | rs2243261 | IL4 | 132012806 | T | ADD | 198 | 1.307 | 0.3863 | 0.6131 | 2.788 | 0.6936 | 0.4879 |
| 5 | rs2243261 | IL4 | 132012806 | T | sex | 198 | 1.032 | 0.3413 | 0.5285 | 2.014 | 0.09134 | 0.9272 |
| 5 | rs2243261 | IL4 | 132012806 | T | AGE | 198 | 1.016 | 0.01127 | 0.9936 | 1.038 | 1.386 | 0.1656 |
| 5 | rs2243261 | IL4 | 132012806 | T | DISTRICT | 198 | 1.059 | 0.3101 | 0.5769 | 1.945 | 0.186 | 0.8524 |
| 5 | rs2243268 | IL4 | 132013963 | C | ADD | 198 | 0.9816 | 0.2234 | 0.6336 | 1.521 | -0.08291 | 0.9339 |
| 5 | rs2243268 | IL4 | 132013963 | C | sex | 198 | 1.049 | 0.3406 | 0.538 | 2.045 | 0.14 | 0.8887 |
| 5 | rs2243268 | IL4 | 132013963 | C | AGE | 198 | 1.016 | 0.01126 | 0.9935 | 1.038 | 1.385 | 0.166 |
| 5 | rs2243268 | IL4 | 132013963 | C | DISTRICT | 198 | 1.046 | 0.3088 | 0.571 | 1.916 | 0.1456 | 0.8842 |
| 5 | rs9282745 | IL4 | 132014000 | A | ADD | 198 | 1.036 | 0.4324 | 0.444 | 2.418 | 0.08239 | 0.9343 |
| 5 | rs9282745 | IL4 | 132014000 | A | sex | 198 | 1.049 | 0.3406 | 0.538 | 2.044 | 0.1397 | 0.8889 |
| 5 | rs9282745 | IL4 | 132014000 | A | AGE | 198 | 1.016 | 0.0113 | 0.9936 | 1.039 | 1.391 | 0.1642 |
| 5 | rs9282745 | IL4 | 132014000 | A | DISTRICT | 198 | 1.04 | 0.3126 | 0.5636 | 1.919 | 0.1256 | 0.9001 |
| 5 | rs73269366 | IL4 | 132018749 | T | ADD | 198 | 1.136 | 0.4591 | 0.4621 | 2.795 | 0.2787 | 0.7805 |
| 5 | rs73269366 | IL4 | 132018749 | T | sex | 198 | 1.053 | 0.3405 | 0.5401 | 2.052 | 0.1507 | 0.8802 |
| 5 | rs73269366 | IL4 | 132018749 | T | AGE | 198 | 1.016 | 0.01131 | 0.9938 | 1.039 | 1.409 | 0.1588 |
| 5 | rs73269366 | IL4 | 132018749 | T | DISTRICT | 198 | 1.028 | 0.3133 | 0.5565 | 1.9 | 0.08919 | 0.9289 |
| 5 | rs3212227 | IL12B | 158742950 | G | ADD | 198 | 0.945 | 0.2176 | 0.6169 | 1.448 | -0.26 | 0.7949 |
| 5 | rs3212227 | IL12B | 158742950 | G | sex | 198 | 1.025 | 0.35 | 0.516 | 2.035 | 0.06987 | 0.9443 |
| 5 | rs3212227 | IL12B | 158742950 | G | AGE | 198 | 1.015 | 0.01133 | 0.9931 | 1.038 | 1.351 | 0.1767 |
| 5 | rs3212227 | IL12B | 158742950 | G | DISTRICT | 198 | 1.046 | 0.3084 | 0.5718 | 1.915 | 0.1474 | 0.8828 |
| 5 | rs2546890 | IL12B | 158759900 | A | ADD | 196 | 1.083 | 0.2408 | 0.6753 | 1.736 | 0.3295 | 0.7418 |
| 5 | rs2546890 | IL12B | 158759900 | A | sex | 196 | 1.006 | 0.3467 | 0.5098 | 1.985 | 0.01696 | 0.9865 |
| 5 | rs2546890 | IL12B | 158759900 | A | AGE | 196 | 1.016 | 0.0113 | 0.9939 | 1.039 | 1.414 | 0.1573 |
| 5 | rs2546890 | IL12B | 158759900 | A | DISTRICT | 196 | 1.046 | 0.3108 | 0.5686 | 1.923 | 0.1433 | 0.886 |
| 6 | rs1736936 | HLAG | 29794317 | G | ADD | 198 | 0.7891 | 0.2187 | 0.514 | 1.211 | -1.083 | 0.2787 |
| 6 | rs1736936 | HLAG | 29794317 | G | sex | 198 | 1.028 | 0.3419 | 0.5262 | 2.01 | 0.08178 | 0.9348 |
| 6 | rs1736936 | HLAG | 29794317 | G | AGE | 198 | 1.016 | 0.01129 | 0.9937 | 1.039 | 1.396 | 0.1628 |
| 6 | rs1736936 | HLAG | 29794317 | G | DISTRICT | 198 | 1.039 | 0.3089 | 0.5672 | 1.904 | 0.1245 | 0.901 |
| 6 | rs1130363 | HLAG | 29797696 | A | ADD | 186 | 0.7078 | 0.2562 | 0.4284 | 1.169 | -1.349 | 0.1773 |
| 6 | rs1130363 | HLAG | 29797696 | A | sex | 186 | 1.158 | 0.3581 | 0.5741 | 2.336 | 0.4101 | 0.6817 |
| 6 | rs1130363 | HLAG | 29797696 | A | AGE | 186 | 1.02 | 0.01165 | 0.9966 | 1.043 | 1.67 | 0.09492 |
| 6 | rs1130363 | HLAG | 29797696 | A | DISTRICT | 186 | 1.056 | 0.3198 | 0.5642 | 1.976 | 0.1701 | 0.865 |
| 6 | rs371194629 | HLAG | 29798581 | ATTTGTTCATGCCT | ADD | 198 | 0.71 | 0.2264 | 0.4555 | 1.106 | -1.513 | 0.1302 |
| 6 | rs371194629 | HLAG | 29798581 | ATTTGTTCATGCCT | sex | 198 | 0.9897 | 0.3445 | 0.5038 | 1.944 | -0.03006 | 0.976 |
| 6 | rs371194629 | HLAG | 29798581 | ATTTGTTCATGCCT | AGE | 198 | 1.016 | 0.01131 | 0.9936 | 1.039 | 1.395 | 0.1629 |
| 6 | rs371194629 | HLAG | 29798581 | ATTTGTTCATGCCT | DISTRICT | 198 | 1.09 | 0.3128 | 0.5904 | 2.012 | 0.2749 | 0.7834 |
| 6 | rs9380142 | HLAG | 29798794 | G | ADD | 187 | 0.9088 | 0.1712 | 0.6498 | 1.271 | -0.5588 | 0.5763 |
| 6 | rs9380142 | HLAG | 29798794 | G | sex | 187 | 0.9366 | 0.3535 | 0.4685 | 1.873 | -0.1852 | 0.853 |
| 6 | rs9380142 | HLAG | 29798794 | G | AGE | 187 | 1.016 | 0.01153 | 0.9935 | 1.039 | 1.392 | 0.1638 |
| 6 | rs9380142 | HLAG | 29798794 | G | DISTRICT | 187 | 1.085 | 0.3157 | 0.5842 | 2.014 | 0.2573 | 0.7969 |
| 6 | rs1610696 | HLAG | 29798803 | G | ADD | 194 | 0.9829 | 0.2366 | 0.6182 | 1.563 | -0.07297 | 0.9418 |
| 6 | rs1610696 | HLAG | 29798803 | G | sex | 194 | 0.967 | 0.3474 | 0.4894 | 1.91 | -0.09661 | 0.923 |
| 6 | rs1610696 | HLAG | 29798803 | G | AGE | 194 | 1.017 | 0.01143 | 0.9942 | 1.04 | 1.455 | 0.1456 |
| 6 | rs1610696 | HLAG | 29798803 | G | DISTRICT | 194 | 0.9853 | 0.3139 | 0.5325 | 1.823 | -0.04717 | 0.9624 |
| 6 | rs1233330 | HLAG | 29799103 | A | ADD | 198 | 0.7394 | 0.4049 | 0.3344 | 1.635 | -0.7458 | 0.4558 |
| 6 | rs1233330 | HLAG | 29799103 | A | sex | 198 | 1.031 | 0.341 | 0.5285 | 2.012 | 0.08988 | 0.9284 |
| 6 | rs1233330 | HLAG | 29799103 | A | AGE | 198 | 1.016 | 0.01128 | 0.994 | 1.039 | 1.428 | 0.1533 |
| 6 | rs1233330 | HLAG | 29799103 | A | DISTRICT | 198 | 1.046 | 0.3087 | 0.571 | 1.915 | 0.1449 | 0.8848 |
| 6 | rs1611139 | HLAG | 29799116 | T | ADD | 187 | 0.7622 | 0.2371 | 0.4789 | 1.213 | -1.145 | 0.2521 |
| 6 | rs1611139 | HLAG | 29799116 | T | sex | 187 | 1.129 | 0.3504 | 0.568 | 2.243 | 0.3459 | 0.7294 |
| 6 | rs1611139 | HLAG | 29799116 | T | AGE | 187 | 1.009 | 0.01173 | 0.9862 | 1.033 | 0.7765 | 0.4375 |
| 6 | rs1611139 | HLAG | 29799116 | T | DISTRICT | 187 | 0.9796 | 0.3211 | 0.5221 | 1.838 | -0.0641 | 0.9489 |
| 6 | rs2517898 | HLAG | 29799746 | G | ADD | 198 | 1.619 | 0.2415 | 1.008 | 2.599 | 1.994 | 0.04616 |
| 6 | rs2517898 | HLAG | 29799746 | G | sex | 198 | 1.029 | 0.3438 | 0.5244 | 2.018 | 0.08201 | 0.9346 |
| 6 | rs2517898 | HLAG | 29799746 | G | AGE | 198 | 1.018 | 0.01147 | 0.9955 | 1.041 | 1.57 | 0.1164 |
| 6 | rs2517898 | HLAG | 29799746 | G | DISTRICT | 198 | 1.047 | 0.3103 | 0.5697 | 1.923 | 0.147 | 0.8831 |
| 6 | rs141206123 | HLAG | 29799849 | C | ADD | 198 | 0.7075 | 0.4466 | 0.2948 | 1.698 | -0.7746 | 0.4386 |
| 6 | rs141206123 | HLAG | 29799849 | C | sex | 198 | 1.077 | 0.3421 | 0.5506 | 2.105 | 0.2159 | 0.829 |
| 6 | rs141206123 | HLAG | 29799849 | C | AGE | 198 | 1.016 | 0.01128 | 0.9939 | 1.039 | 1.418 | 0.1562 |
| 6 | rs141206123 | HLAG | 29799849 | C | DISTRICT | 198 | 1.026 | 0.3085 | 0.5603 | 1.877 | 0.08183 | 0.9348 |
| 6 | rs1136754 | HLAA | 29911921 | 0 | ADD | 197 | NA | NA | NA | NA | NA | NA |
| 6 | rs1136754 | HLAA | 29911921 | 0 | sex | 197 | NA | NA | NA | NA | NA | NA |
| 6 | rs1136754 | HLAA | 29911921 | 0 | AGE | 197 | NA | NA | NA | NA | NA | NA |
| 6 | rs1136754 | HLAA | 29911921 | 0 | DISTRICT | 197 | NA | NA | NA | NA | NA | NA |
| 6 | rs1059563 | HLAA | 29911928 | 0 | ADD | 197 | NA | NA | NA | NA | NA | NA |
| 6 | rs1059563 | HLAA | 29911928 | 0 | sex | 197 | NA | NA | NA | NA | NA | NA |
| 6 | rs1059563 | HLAA | 29911928 | 0 | AGE | 197 | NA | NA | NA | NA | NA | NA |
| 6 | rs1059563 | HLAA | 29911928 | 0 | DISTRICT | 197 | NA | NA | NA | NA | NA | NA |
| 6 | rs1059564 | HLAA | 29911930 | T | ADD | 192 | NA | NA | NA | NA | NA | NA |
| 6 | rs1059564 | HLAA | 29911930 | T | sex | 192 | NA | NA | NA | NA | NA | NA |
| 6 | rs1059564 | HLAA | 29911930 | T | AGE | 192 | NA | NA | NA | NA | NA | NA |
| 6 | rs1059564 | HLAA | 29911930 | T | DISTRICT | 192 | NA | NA | NA | NA | NA | NA |
| 6 | rs1800630 | TNFA | 31542476 | A | ADD | 198 | 0.8595 | 0.3298 | 0.4503 | 1.641 | -0.4589 | 0.6463 |
| 6 | rs1800630 | TNFA | 31542476 | A | sex | 198 | 1.062 | 0.3416 | 0.5436 | 2.074 | 0.1757 | 0.8605 |
| 6 | rs1800630 | TNFA | 31542476 | A | AGE | 198 | 1.016 | 0.0113 | 0.9939 | 1.039 | 1.417 | 0.1566 |
| 6 | rs1800630 | TNFA | 31542476 | A | DISTRICT | 198 | 1.055 | 0.3094 | 0.5753 | 1.935 | 0.1731 | 0.8626 |
| 6 | rs1800629 | TNFA | 31543031 | A | ADD | 198 | 1.295 | 0.2995 | 0.7202 | 2.329 | 0.8636 | 0.3878 |
| 6 | rs1800629 | TNFA | 31543031 | A | sex | 198 | 1.06 | 0.3409 | 0.5432 | 2.067 | 0.1702 | 0.8649 |
| 6 | rs1800629 | TNFA | 31543031 | A | AGE | 198 | 1.015 | 0.01131 | 0.9927 | 1.038 | 1.316 | 0.188 |
| 6 | rs1800629 | TNFA | 31543031 | A | DISTRICT | 198 | 1.07 | 0.3106 | 0.5819 | 1.966 | 0.2164 | 0.8287 |
| 7 | rs2069837 | IL6 | 22768027 | G | ADD | 198 | 1.035 | 0.2878 | 0.5885 | 1.819 | 0.1181 | 0.906 |
| 7 | rs2069837 | IL6 | 22768027 | G | sex | 198 | 1.052 | 0.3422 | 0.5378 | 2.056 | 0.1473 | 0.8829 |
| 7 | rs2069837 | IL6 | 22768027 | G | AGE | 198 | 1.016 | 0.01125 | 0.9936 | 1.038 | 1.391 | 0.1642 |
| 7 | rs2069837 | IL6 | 22768027 | G | DISTRICT | 198 | 1.045 | 0.3083 | 0.571 | 1.912 | 0.1423 | 0.8869 |
| 7 | rs1474347 | IL6 | 22768124 | C | ADD | 198 | 0.5235 | 0.4104 | 0.2342 | 1.17 | -1.577 | 0.1148 |
| 7 | rs1474347 | IL6 | 22768124 | C | sex | 198 | 1.124 | 0.3452 | 0.5712 | 2.211 | 0.3377 | 0.7356 |
| 7 | rs1474347 | IL6 | 22768124 | C | AGE | 198 | 1.016 | 0.01133 | 0.9935 | 1.039 | 1.383 | 0.1667 |
| 7 | rs1474347 | IL6 | 22768124 | C | DISTRICT | 198 | 1.046 | 0.309 | 0.5707 | 1.916 | 0.1448 | 0.8849 |
| 7 | rs2066992 | IL6 | 22768249 | T | ADD | 188 | 1.087 | 0.4806 | 0.4238 | 2.788 | 0.1736 | 0.8622 |
| 7 | rs2066992 | IL6 | 22768249 | T | sex | 188 | 1.078 | 0.3471 | 0.5457 | 2.128 | 0.2151 | 0.8297 |
| 7 | rs2066992 | IL6 | 22768249 | T | AGE | 188 | 1.016 | 0.01137 | 0.9935 | 1.039 | 1.39 | 0.1646 |
| 7 | rs2066992 | IL6 | 22768249 | T | DISTRICT | 188 | 1.05 | 0.3178 | 0.5634 | 1.958 | 0.1545 | 0.8772 |
| 7 | rs1548216 | IL6 | 22769773 | C | ADD | 198 | 0.7308 | 0.2808 | 0.4215 | 1.267 | -1.117 | 0.2641 |
| 7 | rs1548216 | IL6 | 22769773 | C | sex | 198 | 1.029 | 0.3414 | 0.5268 | 2.008 | 0.08238 | 0.9343 |
| 7 | rs1548216 | IL6 | 22769773 | C | AGE | 198 | 1.016 | 0.0113 | 0.9935 | 1.038 | 1.383 | 0.1668 |
| 7 | rs1548216 | IL6 | 22769773 | C | DISTRICT | 198 | 1.072 | 0.3102 | 0.5834 | 1.968 | 0.2228 | 0.8237 |
| 7 | rs2069855 | IL6 | 22772624 | C | ADD | 198 | 0.5796 | 0.8387 | 0.112 | 2.999 | -0.6504 | 0.5155 |
| 7 | rs2069855 | IL6 | 22772624 | C | sex | 198 | 1.051 | 0.3407 | 0.5392 | 2.05 | 0.1471 | 0.8831 |
| 7 | rs2069855 | IL6 | 22772624 | C | AGE | 198 | 1.016 | 0.01129 | 0.9938 | 1.039 | 1.409 | 0.1588 |
| 7 | rs2069855 | IL6 | 22772624 | C | DISTRICT | 198 | 1.063 | 0.3099 | 0.5789 | 1.95 | 0.1957 | 0.8448 |
| 7 | rs1818879 | IL6 | 22772727 | A | ADD | 198 | 0.7397 | 0.3411 | 0.379 | 1.444 | -0.8839 | 0.3768 |
| 7 | rs1818879 | IL6 | 22772727 | A | sex | 198 | 1.054 | 0.3409 | 0.5403 | 2.055 | 0.1539 | 0.8777 |
| 7 | rs1818879 | IL6 | 22772727 | A | AGE | 198 | 1.016 | 0.01124 | 0.9937 | 1.038 | 1.397 | 0.1624 |
| 7 | rs1818879 | IL6 | 22772727 | A | DISTRICT | 198 | 1.017 | 0.3103 | 0.5534 | 1.868 | 0.05308 | 0.9577 |
| 12 | rs2069728 | IFNG | 68547784 | T | ADD | 198 | 0.6445 | 0.2441 | 0.3994 | 1.04 | -1.8 | 0.07191 |
| 12 | rs2069728 | IFNG | 68547784 | T | sex | 198 | 1.001 | 0.3449 | 0.5093 | 1.969 | 0.003827 | 0.9969 |
| 12 | rs2069728 | IFNG | 68547784 | T | AGE | 198 | 1.016 | 0.01137 | 0.9937 | 1.039 | 1.401 | 0.1612 |
| 12 | rs2069728 | IFNG | 68547784 | T | DISTRICT | 198 | 1.058 | 0.3103 | 0.5762 | 1.944 | 0.1828 | 0.8549 |
| 12 | rs2430561 | IFNG | 68552522 | A | ADD | 192 | 1.199 | 0.2757 | 0.6985 | 2.058 | 0.6584 | 0.5103 |
| 12 | rs2430561 | IFNG | 68552522 | A | sex | 192 | 1.062 | 0.3471 | 0.5379 | 2.097 | 0.1731 | 0.8625 |
| 12 | rs2430561 | IFNG | 68552522 | A | AGE | 192 | 1.019 | 0.01145 | 0.9968 | 1.043 | 1.678 | 0.09327 |
| 12 | rs2430561 | IFNG | 68552522 | A | DISTRICT | 192 | 0.9172 | 0.3236 | 0.4864 | 1.729 | -0.2671 | 0.7894 |
| 12 | rs78554979 | IFNG | 68554636 | C | ADD | 198 | 0.6149 | 0.3515 | 0.3088 | 1.225 | -1.383 | 0.1666 |
| 12 | rs78554979 | IFNG | 68554636 | C | sex | 198 | 1.013 | 0.3423 | 0.5177 | 1.981 | 0.03689 | 0.9706 |
| 12 | rs78554979 | IFNG | 68554636 | C | AGE | 198 | 1.017 | 0.01142 | 0.9948 | 1.04 | 1.5 | 0.1335 |
| 12 | rs78554979 | IFNG | 68554636 | C | DISTRICT | 198 | 1.08 | 0.3116 | 0.5866 | 1.99 | 0.2482 | 0.804 |
| 12 | rs2069705 | IFNG | 68555011 | G | ADD | 198 | 1.193 | 0.2246 | 0.7684 | 1.853 | 0.787 | 0.4313 |
| 12 | rs2069705 | IFNG | 68555011 | G | sex | 198 | 1.07 | 0.3417 | 0.548 | 2.091 | 0.1994 | 0.842 |
| 12 | rs2069705 | IFNG | 68555011 | G | AGE | 198 | 1.015 | 0.0113 | 0.9929 | 1.038 | 1.327 | 0.1846 |
| 12 | rs2069705 | IFNG | 68555011 | G | DISTRICT | 198 | 1.063 | 0.3091 | 0.58 | 1.948 | 0.1975 | 0.8434 |
| 16 | rs1801275 | IL4R | 27374400 | A | ADD | 197 | 1.113 | 0.2786 | 0.6445 | 1.921 | 0.3833 | 0.7015 |
| 16 | rs1801275 | IL4R | 27374400 | A | sex | 197 | 1.063 | 0.3409 | 0.5452 | 2.075 | 0.1805 | 0.8567 |
| 16 | rs1801275 | IL4R | 27374400 | A | AGE | 197 | 1.014 | 0.01139 | 0.9913 | 1.037 | 1.193 | 0.2328 |
| 16 | rs1801275 | IL4R | 27374400 | A | DISTRICT | 197 | 1.052 | 0.3093 | 0.5736 | 1.928 | 0.163 | 0.8706 |
| 16 | rs1424241 | HPR | 72078907 | A | ADD | 198 | 0.6591 | 0.3407 | 0.338 | 1.285 | -1.224 | 0.2211 |
| 16 | rs1424241 | HPR | 72078907 | A | sex | 198 | 0.9957 | 0.3436 | 0.5077 | 1.953 | -0.01265 | 0.9899 |
| 16 | rs1424241 | HPR | 72078907 | A | AGE | 198 | 1.015 | 0.01132 | 0.9929 | 1.038 | 1.326 | 0.1847 |
| 16 | rs1424241 | HPR | 72078907 | A | DISTRICT | 198 | 1.084 | 0.311 | 0.589 | 1.993 | 0.2579 | 0.7965 |
| 16 | rs8062041 | HPR | 72088964 | C | ADD | 196 | 1.023 | 0.2052 | 0.6844 | 1.53 | 0.112 | 0.9108 |
| 16 | rs8062041 | HPR | 72088964 | C | sex | 196 | 1.066 | 0.3421 | 0.5454 | 2.085 | 0.1879 | 0.851 |
| 16 | rs8062041 | HPR | 72088964 | C | AGE | 196 | 1.015 | 0.01129 | 0.9927 | 1.038 | 1.315 | 0.1885 |
| 16 | rs8062041 | HPR | 72088964 | C | DISTRICT | 196 | 1.022 | 0.3087 | 0.5581 | 1.872 | 0.07064 | 0.9437 |
| 16 | rs152828 | HPR | 72123886 | T | ADD | 196 | 1.922 | 1.427 | 0.1171 | 31.54 | 0.4577 | 0.6471 |
| 16 | rs152828 | HPR | 72123886 | T | sex | 196 | 1.078 | 0.3421 | 0.5511 | 2.107 | 0.2183 | 0.8272 |
| 16 | rs152828 | HPR | 72123886 | T | AGE | 196 | 1.017 | 0.01136 | 0.9947 | 1.04 | 1.495 | 0.1349 |
| 16 | rs152828 | HPR | 72123886 | T | DISTRICT | 196 | 1.069 | 0.3106 | 0.5817 | 1.965 | 0.2156 | 0.8293 |
| 19 | rs375947 | IL12RB1 | 18180451 | 0 | ADD | 198 | NA | NA | NA | NA | NA | NA |
| 19 | rs375947 | IL12RB1 | 18180451 | 0 | sex | 198 | NA | NA | NA | NA | NA | NA |
| 19 | rs375947 | IL12RB1 | 18180451 | 0 | AGE | 198 | NA | NA | NA | NA | NA | NA |
| 19 | rs375947 | IL12RB1 | 18180451 | 0 | DISTRICT | 198 | NA | NA | NA | NA | NA | NA |
| 19 | rs11575934 | IL12RB1 | 18186618 | C | ADD | 197 | 1.142 | 0.377 | 0.5455 | 2.391 | 0.3521 | 0.7248 |
| 19 | rs11575934 | IL12RB1 | 18186618 | C | sex | 197 | 1.078 | 0.3431 | 0.5504 | 2.112 | 0.2198 | 0.826 |
| 19 | rs11575934 | IL12RB1 | 18186618 | C | AGE | 197 | 1.017 | 0.01134 | 0.995 | 1.04 | 1.514 | 0.13 |
| 19 | rs11575934 | IL12RB1 | 18186618 | C | DISTRICT | 197 | 1.056 | 0.3095 | 0.5758 | 1.937 | 0.1761 | 0.8602 |
| 22 | rs12483859 | MIF | 24234807 | T | ADD | 198 | 1.329 | 0.2314 | 0.8447 | 2.093 | 1.231 | 0.2185 |
| 22 | rs12483859 | MIF | 24234807 | T | sex | 198 | 1.05 | 0.3417 | 0.5372 | 2.05 | 0.1414 | 0.8875 |
| 22 | rs12483859 | MIF | 24234807 | T | AGE | 198 | 1.013 | 0.01152 | 0.9902 | 1.036 | 1.105 | 0.2691 |
| 22 | rs12483859 | MIF | 24234807 | T | DISTRICT | 198 | 1.022 | 0.3097 | 0.5569 | 1.875 | 0.07013 | 0.9441 |
| 22 | rs36086171 | MIF | 24235455 | G | ADD | 189 | 0.5345 | 0.2499 | 0.3275 | 0.8723 | -2.507 | 0.01219 |
| 22 | rs36086171 | MIF | 24235455 | G | sex | 189 | 1.257 | 0.3537 | 0.6284 | 2.514 | 0.6464 | 0.518 |
| 22 | rs36086171 | MIF | 24235455 | G | AGE | 189 | 1.008 | 0.01172 | 0.9849 | 1.031 | 0.666 | 0.5054 |
| 22 | rs36086171 | MIF | 24235455 | G | DISTRICT | 189 | 0.9771 | 0.3207 | 0.5212 | 1.832 | -0.07213 | 0.9425 |
| 22 | rs9282783 | MIF | 24236359 | G | ADD | 198 | 1.005 | 0.4309 | 0.432 | 2.339 | 0.01203 | 0.9904 |
| 22 | rs9282783 | MIF | 24236359 | G | sex | 198 | 1.047 | 0.3403 | 0.5375 | 2.04 | 0.1356 | 0.8921 |
| 22 | rs9282783 | MIF | 24236359 | G | AGE | 198 | 1.016 | 0.01132 | 0.9935 | 1.039 | 1.383 | 0.1667 |
| 22 | rs9282783 | MIF | 24236359 | G | DISTRICT | 198 | 1.044 | 0.3085 | 0.5704 | 1.912 | 0.1406 | 0.8882 |
| 22 | rs11548056. | MIF | 24237053 | 0 | ADD | 197 | NA | NA | NA | NA | NA | NA |
| 22 | rs11548056. | MIF | 24237053 | 0 | sex | 197 | NA | NA | NA | NA | NA | NA |
| 22 | rs11548056. | MIF | 24237053 | 0 | AGE | 197 | NA | NA | NA | NA | NA | NA |
| 22 | rs11548056. | MIF | 24237053 | 0 | DISTRICT | 197 | NA | NA | NA | NA | NA | NA |
| 22 | rs2000466 | MIF | 24237862 | G | ADD | 193 | 1.625 | 0.2271 | 1.041 | 2.536 | 2.137 | 0.03258 |
| 22 | rs2000466 | MIF | 24237862 | G | sex | 193 | 1.066 | 0.3469 | 0.5403 | 2.105 | 0.1853 | 0.853 |
| 22 | rs2000466 | MIF | 24237862 | G | AGE | 193 | 1.019 | 0.01169 | 0.9959 | 1.043 | 1.612 | 0.107 |
| 22 | rs2000466 | MIF | 24237862 | G | DISTRICT | 193 | 1.063 | 0.313 | 0.5754 | 1.962 | 0.1939 | 0.8462 |
| 22 | rs34383331 | MIF | 24238079 | A | ADD | 196 | 1.599 | 0.2549 | 0.9701 | 2.634 | 1.841 | 0.06564 |
| 22 | rs34383331 | MIF | 24238079 | A | sex | 196 | 1.092 | 0.3455 | 0.5548 | 2.149 | 0.2548 | 0.7988 |
| 22 | rs34383331 | MIF | 24238079 | A | AGE | 196 | 1.018 | 0.0114 | 0.9954 | 1.041 | 1.555 | 0.1199 |
| 22 | rs34383331 | MIF | 24238079 | A | DISTRICT | 196 | 1.03 | 0.3109 | 0.5602 | 1.895 | 0.09622 | 0.9233 |
| 22 | rs136174 | APOL1 | 36661536 | C | ADD | 194 | 1.101 | 0.6782 | 0.2914 | 4.161 | 0.1421 | 0.887 |
| 22 | rs136174 | APOL1 | 36661536 | C | sex | 194 | 1.012 | 0.3459 | 0.5137 | 1.993 | 0.03418 | 0.9727 |
| 22 | rs136174 | APOL1 | 36661536 | C | AGE | 194 | 1.017 | 0.0114 | 0.9947 | 1.04 | 1.492 | 0.1356 |
| 22 | rs136174 | APOL1 | 36661536 | C | DISTRICT | 194 | 1.019 | 0.3104 | 0.5545 | 1.872 | 0.05979 | 0.9523 |
| 22 | rs73885316 | APOL1 | 36661674 | A | ADD | 198 | 1.11 | 0.939 | 0.1762 | 6.992 | 0.1112 | 0.9114 |
| 22 | rs73885316 | APOL1 | 36661674 | A | sex | 198 | 1.048 | 0.3403 | 0.5381 | 2.042 | 0.1388 | 0.8896 |
| 22 | rs73885316 | APOL1 | 36661674 | A | AGE | 198 | 1.016 | 0.01132 | 0.9933 | 1.038 | 1.37 | 0.1706 |
| 22 | rs73885316 | APOL1 | 36661674 | A | DISTRICT | 198 | 1.045 | 0.3083 | 0.571 | 1.912 | 0.1425 | 0.8867 |
| 22 | rs136177 | APOL1 | 36661842 | G | ADD | 194 | 1.179 | 0.515 | 0.4297 | 3.235 | 0.3196 | 0.7492 |
| 22 | rs136177 | APOL1 | 36661842 | G | sex | 194 | 0.9988 | 0.3453 | 0.5077 | 1.965 | -0.00361 | 0.9971 |
| 22 | rs136177 | APOL1 | 36661842 | G | AGE | 194 | 1.018 | 0.01141 | 0.9953 | 1.041 | 1.547 | 0.1219 |
| 22 | rs136177 | APOL1 | 36661842 | G | DISTRICT | 194 | 1.01 | 0.3109 | 0.5494 | 1.858 | 0.03305 | 0.9736 |
| 22 | rs73885319 | APOL1_G1 | 36661906 | G | ADD | 198 | 1.136 | 0.3434 | 0.5795 | 2.226 | 0.371 | 0.7106 |
| 22 | rs73885319 | APOL1_G1 | 36661906 | G | sex | 198 | 1.043 | 0.3405 | 0.5349 | 2.032 | 0.1226 | 0.9024 |
| 22 | rs73885319 | APOL1_G1 | 36661906 | G | AGE | 198 | 1.016 | 0.01126 | 0.9937 | 1.039 | 1.398 | 0.1622 |
| 22 | rs73885319 | APOL1_G1 | 36661906 | G | DISTRICT | 198 | 1.054 | 0.3099 | 0.5742 | 1.934 | 0.1694 | 0.8655 |
| 22 | rs143830837 | APOL1 | 36662042 | 0 | ADD | 188 | NA | NA | NA | NA | NA | NA |
| 22 | rs143830837 | APOL1 | 36662042 | 0 | sex | 188 | NA | NA | NA | NA | NA | NA |
| 22 | rs143830837 | APOL1 | 36662042 | 0 | AGE | 188 | NA | NA | NA | NA | NA | NA |
| 22 | rs143830837 | APOL1 | 36662042 | 0 | DISTRICT | 188 | NA | NA | NA | NA | NA | NA |
| 22 | rs71785313 | APOL1_G2 | 36662046 | DEL | ADD | 196 | 0.2045 | 0.4328 | 0.08758 | 0.4777 | -3.667 | 0.000246 |
| 22 | rs71785313 | APOL1_G2 | 36662046 | DEL | sex | 196 | 0.9858 | 0.3531 | 0.4935 | 1.97 | -0.0404 | 0.9678 |
| 22 | rs71785313 | APOL1_G2 | 36662046 | DEL | AGE | 196 | 1.021 | 0.01179 | 0.9973 | 1.044 | 1.73 | 0.08364 |
| 22 | rs71785313 | APOL1_G2 | 36662046 | DEL | DISTRICT | 196 | 1.062 | 0.3228 | 0.5643 | 2 | 0.1876 | 0.8512 |

Table showing analysis from the logistic regression. There are four rows for each SNP one for the main effect of HAT on the row named “ADD” then a row for the effect of each covariate (sex, AGE, DISTRICT). The results are very similar to the Fisher exact test. There is no evidence for an effect on APOL1_G2 of sex (p=0.97), age (p= 0.084) or district of origin (0.85)
